# Versorgung und Krankheitskosten der Urtikaria bei Kindern in Deutschland: Arzneimittel, ärztliche und stationäre Leistungen

**DOI:** 10.1007/s00105-024-05346-3

**Published:** 2024-05-22

**Authors:** Petra Staubach, Caroline Mann, Kristina Hagenström, Matthias Augustin

**Affiliations:** 1 Klinik und Poliklinik für Dermatologie, Universitätsmedizin Mainz, Mainz, Deutschland; 2 Institut für Versorgungsforschung in der Dermatologie und bei Pflegeberufen (IVDP), Universitätsklinikum Hamburg-Eppendorf (UKE), Hamburg, Deutschland

**Keywords:** Chronische Urtikaria, Kostenfaktoren, Therapiekosten, Systemische Arzneimittel, Topische Arzneimittel, Chronic urticaria, Medical cost factors, Treatment costs, Systemic drug administration, Topical drug administration

## Abstract

**Hintergrund:**

Daten zu den Verlaufsformen der Urtikaria bei Kindern existieren, es mangelt jedoch an fundierten Daten, um eine qualitativ hochwertige Versorgung zu gewährleisten.

**Methode:**

Es erfolgte eine retrospektive Sekundärdatenanalyse bei Kindern mit Urtikaria in den Routinedaten einer deutschen Krankenversicherung (DAK-Gesundheit). Eingeschlossen wurden Versicherte unter 18 Jahren, die 2010 bis 2015 ambulant oder stationär mit Urtikariadiagnose (gemäß ICD-10-Klassifikation) behandelt wurden. Statistische Vergleiche wurden nach Adjustierung von Alter und Geschlecht gegenüber Personen ohne Urtikariadiagnose vorgenommen.

**Ergebnisse:**

Im Jahr 2015 wiesen unter 151.248 minderjährigen Versicherten 1904 (1,3 %) eine Urtikariadiagnose auf; 70,9 % der Kinder mit Urtikaria besuchten mindestens einen ambulanten Arzt, von diesen Visiten fanden 70,9 % beim Kinderarzt, 52,5 % beim Allgemeinmediziner und 33,0 % beim Dermatologen statt; 11 % wurden stationär behandelt; 72,9 % der Kinder und Jugendlichen mit Urtikaria vs. 28,9 % ohne wurden topisch oder systemisch behandelt, darunter 10,5 % vs. 2,6 % mit topischen Therapien und 70,0 % vs. 27,5 % mit Systemtherapien. Die am häufigsten eingesetzten oralen Medikamente bei Urtikaria waren Cetirizin (44,2 %), Prednisolon (9,8 %) und Dimetinden (2,0 %). Topisch wurde am häufigsten Methylprednisolonaceponat (49,8 %) verordnet. Die Therapiekosten für systemische Arzneimittel betrugen 24,00 € pro Patient, die für topische 1,58 € pro Patient.

**Diskussion:**

Die Versorgung der chronischen Urtikaria im Kindes- und Jugendalter wird im Wesentlichen durch Kinder‑, Haus- und Hautärzte getragen. Systemische und topische Arzneimittel sowie stationäre Leistungen stellen die wichtigsten Kostenfaktoren dar.

**Zusatzmaterial online:**

Die Online-Version dieses Beitrags (10.1007/s00105-024-05346-3) enthält 5 zusätzliche Tabellen. Beitrag und Zusatzmaterial stehen Ihnen im elektronischen Volltextarchiv auf https://www.springermedizin.de/die-dermatologie zur Verfügung. Sie finden das Zusatzmaterial am Beitragsende unter „Supplementary Information“.

## Abstract (en)

**Background:**

Data on the course of urticaria in children exist, but there is a lack of sound data on patient management to ensure high-quality care.

**Methods:**

Retrospective secondary data analysis in the field of health care and epidemiology in children with urticaria based on routine data from a German health insurance company (DAK-Gesundheit). Data from insured persons under 18 years of age who were treated as outpatients or inpatients with a diagnosis of urticaria (according to ICD-10 classification) in 2010–2015 were included. The control group consisted of children without a corresponding diagnosis, in order to clarify health economic and care-related differences after adjusting for age and gender.

**Results:**

In 2015, 1904 (1.3%) of 151,248 insured minors had a diagnosis of urticaria. Of the children with urticaria, 70.9% visited at least one physician on an outpatient basis. Of these visits, 70.9% were made to a pediatrician, 52.5% to a general practitioner and 33.0% to a dermatologist; 11% were treated as inpatients. With a total of 151,248 insured persons, 1904 of whom were diagnosed with urticaria, 72.9% of children and adolescents with versus 28.9% without urticaria were treated topically or systemically in 2015, including 10.5% of children with urticaria vs. 2.6% without urticaria received topical therapy and 70.0% with urticaria received systemic therapy vs. 27.5% without urticaria with systemic therapy. The most commonly used oral medications for urticaria were cetirizine (44.2%), prednisolone (9.8%), and dimetindene (2.0%) . Topical methylprednisolone aceponate (49.8%) was prescribed most frequently. The therapy costs for systemic drugs was € 24.00 per patient, while topical drugs cost € 1.58 per patient.

**Conclusion:**

The lack of guidelines for the standardization of treatment in children still leads to ambiguities and different treatment concepts among the specialist groups, which must be eliminated in order to enable more efficient therapies. The treatment of chronic urticaria in children and adolescents is mainly carried out by pediatricians, general practitioners and dermatologists. Systemic and topical medications as well as inpatient services are the most important cost factors.

Die chronische Urtikaria bei Kindern wird in der medizinischen Fachliteratur nur selten adressiert, obwohl sie mit einer relevanten Krankheitslast verbunden sein kann [1, 3]. Insbesondere Daten zur Versorgung bei Kindern und zu den Kosten für medizinische Leistungen wurden bisher nicht publiziert.

In einer früheren Datenanalyse wurde die Prävalenz der Urtikaria bei Kindern im Alter von 0 bis 18 Jahren in Deutschland mit 1,7 % beschrieben [4]. Diese Prävalenz nahm in der Altersgruppe der 0‑ bis 3‑Jährigen von 3,0 % auf 1,0 % in der Altersgruppe der 14- bis 18-Jährigen ab. Jungen und Mädchen waren in allen Altersgruppen fast gleich häufig betroffen. Komorbiditäten des atopischen Formenkreises traten bei Kindern mit Urtikaria häufiger auf als in der Kontrollgruppe (16,0 % vs. 8,0 %) [4].

Während für Erwachsene klinische Informationen, Studien und Behandlungsleitlinien verfügbar sind, wurden Kinder mit Urtikaria nicht systematisch untersucht. Die aktuellen Leitlinien erwähnen Kinder und verweisen auf individualisierte, nicht standardisierte und sogar neuartige Therapieansätze [9, 15].

Ziel der vorliegenden Studie war es, die Versorgungssituation von Kindern mit Urtikaria unter den folgenden Forschungsfragen zu analysieren:

1. Wie sind die Strukturen und Prozesse der ambulanten und stationären Versorgung bei Urtikaria im Kindesalter?
2. Welche Arzneimittel werden eingesetzt?
3. Welche Verordnungen erfolgen in Abhängigkeit von den Verordnern?
4. Welche Kosten resultieren aus der Arzneimittelversorgung?
5. Welche Unterschiede finden sich hinsichtlich dieser Punkte zu Kindern ohne Urtikaria?
6. Welche Rückschlüsse lassen sich hieraus auf die Qualität der Versorgung ziehen?

## Methoden

### Studiendesign und Studienpopulation

Retrospektive Sekundärdatenanalyse mit Daten einer repräsentativen Stichprobe von 40 % der Mitglieder der DAK-Gesundheit (DAK-G), einer deutsche Krankenkasse mit 5,8 Mio. Mitgliedern im Jahr 2010 (Zensusstichtag 31.12.2010 – Gesamtkollektiv aller Versicherten einschließlich Familienangehöriger; 61 % weiblich, Durchschnittsalter 47,4 Jahre; *N* = 2.319.584). Die DAK‑G ist eine bundesweit vertretene gesetzliche Krankenversicherung (GKV), in der ca. 7,1 % der deutschen Bevölkerung versichert sind. Im Jahr 2020 waren ca. 90 % der deutschen Bevölkerung (ca. 73 Mio.) Mitglied in einer der 105 Krankenkassen; der restliche Teil der Bevölkerung war privat versichert [4]. Diese Versichertenstichprobe wurde von der DAK‑G anonymisiert und dem Institut zur Verfügung gestellt. Diese Gruppe durchgängig Versicherter wurde über 6 Jahre (2010 bis 2015) beobachtet.

### Falldefinition

Für die vorliegenden Analysen wurde aus der vorliegenden DAK-Stichprobe eine prävalente Kohorte gebildet. Diese Kohorte umfasst DAK-Versicherte unter 18 Jahren, die am Stichtag 31.12.2015 versichert waren (*n* = 151.248). Als Fälle von Urtikaria berücksichtigt wurden alle Kinder mit mindestens einer ambulanten, ärztlich gesicherten oder als Krankenhausdiagnose (Neben- oder Hauptentlassungsdiagnose) kodierten Urtikaria im Jahr 2015 gemäß der International Statistical Classification of Diseases and Related Health Problems (ICD-10) (L50.0 bis L50.9).

#### Ambulant-ärztliche Versorgung sowie stationäre Versorgung

Für die Erhebung der ambulanten Inanspruchnahme der Versichertenkohorte wurden alle Arztbesuche im Beobachtungsjahr 2015 herangezogen. Die behandelnden Facharztgruppen (Allgemeinmediziner, Dermatologen, Kinderärzte) wurden anhand der Facharztgruppenschlüssel identifiziert [2].

Für die Erhebung der stationären Versorgung der prävalenten Versichertenkohorte wurden alle stationären Aufenthalte im Jahr 2015 aufgrund von Urtikaria berücksichtigt.

#### Arzneimittelinanspruchnahme und Behandlungsverläufe

Alle relevanten Arzneimittelverordnungen für Urtikaria werden entsprechend ihrer anatomisch-therapeutischen-chemischen (ATC) und definierten Tagesdosis(DDD)-Klassifikation [3] berücksichtigt (Online-Tab. 1 und 2). Wie in der Leitlinie wurde keine Unterscheidung zwischen zugelassenen und nichtzugelassenen Arzneimitteln getroffen [14]. Die individuellen Ergebnisse wurden nach Art und Anzahl (nach DDD) der Verordnungen dargestellt.

Analysiert wurde die medikamentöse Behandlungshistorie von inzident erkrankten Kindern in Bezug auf Veränderungen der DDD-Menge relevanter Arzneimittelgruppen (Tab. 1, Online-Tab. 3). Inzidente Patienten wurden als Versicherte definiert, die im Jahr 2010 keine Urtikariadiagnose und in den Jahren 2011 bis 2013 mindestens eine Urtikariadiagnose (ambulant bestätigt oder Krankenhausdiagnose [Hauptentlassung oder Nebendiagnose]) erhielten.

**Tab. 1:** Anteil an stationären Aufenthalten und mittlere Aufenthaltsdauer bei Kindern mit einer Urtikariadiagnose im Jahr 2015 (*n* = 1904) im Vergleich zu nicht an Urtikaria erkrankten Kindern (*n* = 149.344)

Geschlecht	Altersklassen	Versicherte *n* (%)	MW-Aufenthalte^a^	Min-Max-Aufenthalte	Aufenthaltsdauer (Tage)	Min-Max-Aufenthaltsdauer (Tage)
**Versicherte mit Urtikaria**						
*Gesamt*	Gesamt	203 (10,66)	1,29	1–11	8,16	1–101
	0 bis unter 3 J	0 (0,00)	0	0–0	0	0–0
	3 bis unter 7 J	12 (0,63)	1,17	1–2	5,67	2–16
	7 bis unter 11 J	66 (3,47)	1,30	1–7	6,65	1–71
	11 bis unter 14 J	40 (2,10)	1,20	1–2	8,88	1–101
	14 bis unter 18 J	85 (4,46)	1,34	1–11	9,34	1–86
*Jungen*	Gesamt	87 (4,57)	1,38	1–11	8,40	1–86
*Mädchen*	Gesamt	116 (6,09)	1,22	1–4	7,97	1–101
**Versicherte ohne Urtikaria**						
*Gesamt*	Gesamt	8411 (5,63)	1,28	1–18	10,36	1–332
	0 bis unter 3 J	0 (0,00)	0	0–0	0	0–0
	3 bis unter 7 J	449 (0,30)	1,21	1–16	6,03	1–128
	7 bis unter 11 J	1888 (1,26)	1,23	1–16	8,14	1–250
	11 bis unter 14 J	1953 (1,31)	1,25	1–10	9,76	1–181
	14 bis unter 18 J	4121 (2,76)	1,33	1–18	12,13	1–332
*Jungen*	Gesamt	4192 (2,81)	1,26	1–16	9,35	1–250
*Mädchen*	Gesamt	4219 (2,83)	1,30	1–18	11,37	1–332

*J* Jahre

^a^Mittlere Anzahl der stationären Aufenthalte

#### Krankheitskosten

Bei der Ermittlung der Kosten für ambulant verordnete Arzneimittel wurden die reinen Kosten aus den Routinedaten für die prävalente Kohorte übernommen (Online-Tab. 3 und 4).

### Statistische Analyse

Kategoriale Variablen wurden in Prozent dargestellt, für kontinuierliche Variablen wurden der Median und die Spannweite verwendet. Die Einteilung der Altersgruppen folgte der Systematik des bundesweiten Kinder- und Jugendgesundheitssurvey [10]. Das Alter wurde aus dem Stichtag 31.12.2015 und dem Geburtsjahr berechnet. Die Arzneimittelkosten wurden aus Kostenträgerperspektive dargestellt, als Bruttobeträge ohne Abzüge der Mehrwertsteuer und der Herstellerrabatte. Die Analysen erfolgten mit SAS Version 9.4 deutsch (SAS Institute, Cary, North Carolina, USA).

## Ergebnisse

### Studienpopulation

Zum Stichtag 31.12.2015 waren 151.248 Personen unter 18 Jahren bei der DAK versichert, darunter 74.111 (49,0 %) Mädchen und 77.137 (51,0 %) Jungen. Bei 1904 dieser Versicherten wurde im Jahr 2015 mindestens 1‑mal die gesicherte Diagnose Urtikaria kodiert, was einer administrativen Prävalenz von ca. 1,25 % entspricht [11].

### Ambulante und stationäre Versorgung

Etwa 70,9 % der Versicherten suchten im Beobachtungsjahr wegen der Urtikaria mindestens 1‑mal einen Kinderarzt auf, 52,5 % einen Allgemeinmediziner und 33,0 % einen Dermatologen (Online-Tab. 3). Kinder mit Urtikaria suchten diese Fachärzte häufiger auf als Kinder ohne Urtikaria, beispielsweise 33,0 % vs. 13,7 % einen Dermatologen.

Insgesamt wiesen knapp 11 % der Kinder mit einer Urtikaria-Codierung mindestens einen stationären Aufenthalt auf (Tab. 1) im Vergleich zu 6 % der Kinder gleichen Alters ohne entsprechende Diagnose. Die höchste mittlere Anzahl an Krankenhausaufenthalten fand sich in der Altersgruppe 7 bis unter 11 Jahre (1,3 Aufenthalte) im Vergleich zu der vergleichbaren Altersgruppe ohne Urtikaria (1,2 Aufenthalte). Zudem werden Mädchen mit Urtikaria häufiger stationär behandelt (6,1 %) als Mädchen ohne Urtikaria (2,8 %).

### Arzneimittelversorgung

Insgesamt wurden im Jahr 2015 bei 1387 (72,9 %) der versicherten Kinder und Jugendlichen mit Urtikaria 3123 Arzneimittelverordnungen abgerechnet (Tab. 2) Durchschnittlich entfielen dabei 2,3 Verordnungen mit 39 DDD auf jeden Versicherten.

**Tab. 2:** Arzneimittelverordnungen bei Kindern und Jugendlichen mit (*n* = 1904) und ohne Urtikaria im Jahr 2015 (*n* = 149.344)

Arzneimittel	Versicherte *n* (%)	Verordnungen	DDD	Verordnungen pro Versicherten	DDD pro Versicherten
**Mit Urtikaria**					
*Gesamt*	1387 (72,85)	3123	54.165	2,25	39
*Systemisch*	1333 (70,01)	2900	48.910	2,18	37
Antibiotika zur systemischen Anwendung	724 (54,31)	1275	11.639	1,76	16
Cetirizin	589 (44,19)	743	11.984	1,26	20
Penicilline	334 (25,06)	453	4696	1,36	14
Dimetinden	280 (21,01)	328	1918	1,17	7
Desloratadin	135 (10,13)	212	7831	1,57	58
Rupatadin	50 (3,75)	71	2242	1,42	45
Levocetirizin	24 (1,80)	36	878	1,50	37
Ebastin	18 (1,35)	20	1040	1,11	58
Antihistaminika zur systemischen Anwendung	6 (0,45)	6	55	1,00	9
Ranitidin	3 (0,23)	13	296	4,33	99
Dexamethason	51 (3,83)	62	662	1,22	13
Methylprednisolon	2 (0,15)	3	64	1,50	32
Prednisolon	133 (9,98)	165	5605	1,24	42
*Topisch*	199 (10,45)	223	5255	1,12	26
Methylprednisolonaceponat	99 (49,75)	108	3965	1,09	40
Antihistaminika zur topischen Anwendung	79 (39,70)	81	1290	1,03	16
**Ohne Urtikaria**					
*Gesamt*	43.097 (28,86)	146.072	1.559.112	1,90	20
*Systemisch*	41.133 (27,54)	89.956	1.042.681	2,19	25
Antibiotika zur systemischen Anwendung	35.751 (86,92)	56.116	516.432	1,57	14
Cetirizin	4344 (10,56)	5911	117.191	1,36	27
Penicilline	15.596 (37,92)	19.957	215.420	1,28	14
Dimetinden	1530 (3,72)	1707	10.428	1,12	7
Desloratadin	1020 (2,48)	1570	47.265	1,54	46
Rupatadin	120 (0,29)	141	2496	1,18	21
Levocetirizin	282 (0,69)	415	15.103	1,47	54
Ebastin	127 (0,31)	182	7950	1,43	63
Antihistaminika zur systemischen Anwendung	28 (0,07)	40	635	1,43	23
Ranitidin	120 (0,29)	141	2496	1,18	21
Dexamethason	1007 (2,45)	1216	18.393	1,21	18
Methylprednisolon	89 (0,22)	124	5279	1,39	59
Prednisolon	1813 (4,41)	2436	83.595	1,34	46
*Topisch*	3819 (2,56)	3393	108.582	0,89	28
Methylprednisolonaceponat	2134 (55,88)	2636	97.300	1,24	46
Antihistaminika zur topischen Anwendung	710 (18,59)	757	11.282	1,07	16

*DDD* „defined daily dose“ (definierte Tagesdosis)

Mehrfachverordnungen sind möglich

Insgesamt 10,5 % der Kinder und Jugendlichen mit Urtikaria erhielten im Jahr 2015 ein topisches Arzneimittel, und 70,0 % wurden mit einem systemisch wirksamen Arzneimittel behandelt. Die systemischen Arzneimitteltherapien machen mit 48.910 DDD einen Anteil von 90,2 % der gesamten Arzneimittelversorgung dieser Patienten aus. Von den systemischen Arzneimitteln erhielt die Mehrheit der Kinder und Jugendlichen mit Urtikaria Antibiotika (54,3 % mit 1275 Verordnungen), Cetirizin (44,2 % mit 743 Verordnungen), gefolgt von Penicillinen (25,1 % mit 453 Verordnungen) und Dimetinden (21,0 % mit 328 Verordnungen). Von den topischen Arzneimitteln wurde Methylprednisolonaceponat am häufigsten bei Kindern und Jugendlichen mit Urtikaria verordnet (49,8 % mit 108 Verordnungen).

Im Jahr 2015 erhielten insgesamt 2,7 % der Kinder und Jugendlichen mit Urtikaria und einer Arzneimitteltherapie eine Kombinationstherapie, bestehend aus der Verschreibung eines nicht sedierenden Antihistaminikums und eines weiteren Arzneimittels (Online-Tab. 5); 4,3 % erhielten hingegen eine Monotherapie, wovon 93,3 % topische und 7,1 % systemische Arzneimittel einschlossen.

Unter den Kindern und Jugendlichen mit Urtikaria, die 2015 einen Kinderarzt aufsuchten, erhielten 58 % ein systemisches Arzneimittel (58,0 % mit 1477 Verordnungen und 19.875 DDD). Demgegenüber erhielten 22,9 % derjenigen, die einen Dermatologen aufsuchten, ein systemisches Arzneimittel (222 Verordnungen und 6606 DDD) (Tab. 3).

**Tab. 3:** Arzneimittelverordnungen bei Kindern und Jugendlichen mit und ohne Urtikaria nach Facharztgruppe im Jahr 2015

Facharztbezeichnung	Arzneimittelgruppe	Versicherte *n* (%)	Verordnungen	DDD	Verordnungen pro Versicherten	DDD pro Versicherten
**Mit Urtikaria**						
*Allgemeinmedizin*	Systemisch	453 (45,35)	760	11.245	1,68	25
	Topisch	47 (4,70)	51	1773	1,09	38
*Dermatologie*	Systemisch	144 (22,89)	222	6606	1,54	46
	Topisch	44 (7,00)	44	1767	1,00	40
*Hals-Nasen-Ohrenheilkunde*	Systemisch	68 (17,80)	87	880	1,28	13
	Topisch	0 (0,00)	0	0	0,00	0
*Innere Medizin*	Systemisch	21 (22,34)	32	1132	1,52	54
	Topisch	2 (2,13)	2	53	1,00	27
*Pädiatrie*	Systemisch	783 (58,04)	1477	19.875	1,89	25
	Topisch	91 (6,75)	102	2028	1,12	22
**Ohne Urtikaria**						
*Allgemeinmedizin*	Systemisch	17.984 (26,84)	26.363	272.451	1,47	15
	Topisch	953 (1,42)	1138	35.642	1,19	37
*Dermatologie*	Systemisch	1076 (5,26)	1497	31.553	1,39	29
	Topisch	954 (4,67)	1129	44.778	1,18	47
*Hals-Nasen-Ohrenheilkunde*	Systemisch	3569 (17,13)	4608	54.944	1,29	15
	Topisch	82 (0,39)	93	1615	1,13	20
*Innere Medizin*	Systemisch	361 (9,29)	449	9895	1,24	27
	Topisch	2 (0,05)	2	53	1,00	27
*Pädiatrie*	Systemisch	17.890 (21,94)	27.640	340.664	1,54	19
	Topisch	1444 (1,77)	1720	43.787	1,19	30

*DDD* „defined daily dose“ (definierte Tagesdosis)

### Arzneimittelbehandlungsverläufe

**Abb. 1:**
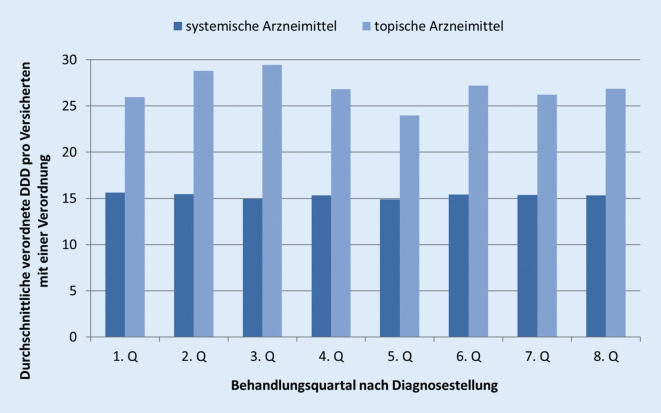
„Defined daily dose“ (DDD, definierte Tagesdosis) verordneter systemischer und topischer Arzneimittel pro inzidenten Versicherten mit Urtikaria im Verlauf von 8 Quartalen ab Erstdiagnose (*n* = 9636)

Bei der Analyse der DDD systemischer Arzneimittel fanden sich im Verlauf von 2 Jahren nur geringe Schwankungen über die Behandlungsquartale hinweg: Im ersten Behandlungsquartal waren dies 16 DDDs pro Versicherten, anschließend relativ konstant um 15 DDDs pro Versicherten (Abb. 1).

Bei den topischen Arzneimitteln ist eine leichte Wellenform zu erkennen. Im ersten Quartal (Diagnosequartal) wurden 26 DDDs pro Versicherten verordnet. Bis zum dritten Quartal stieg die Anzahl auf 29 DDDs pro Versicherten und fiel im fünften Quartal auf 24 DDDs pro Versicherten. Im achten Quartal wurden 27 DDDs pro Versicherten verschrieben.

### Arzneimittelkosten

Die Gesamtkosten für Arzneimittel bei Kindern mit Urtikaria beliefen sich 2015 auf 48.720 € (Online-Tab. 4). Systemische Arzneimittel verursachten mit 45.704 € (24 €/Patient) die höchsten Kosten. Für topische Medikamente beliefen sich die Gesamtkosten auf 3016 €. Die Kosten pro Versicherten für Arzneimittel waren bei Mädchen mit Urtikaria (13,53 €) etwas höher als bei Jungen mit Urtikaria (12,06 €).

## Diskussion

Das Ziel dieser Studie war die Analyse der Versorgung von Kindern und Jugendlichen in Deutschland, die an chronischer Urtikaria leiden. Weiterhin wurden die damit verbundenen Krankheitskosten erfasst. Da weltweit nur wenige Daten zu dieser Erkrankung vorliegen, ist diese Studie von großer Bedeutung für die Versorgung von Betroffenen. Es wird Bezug genommen auf eine parallele Analyse der administrativen Prävalenz und Inzidenz der chronischen Urtikaria bei Kindern derselben Kohorte, die eine Häufigkeit von 1,7 % unter insgesamt 313.581 (mit)versicherten Kindern in Deutschland aufwies. Die Prävalenz war in der Altersgruppe der 0‑ bis 3‑Jährigen mit 3,0 % höher als in der Altersgruppe der 14- bis 18-Jährigen mit 1,0 %. Jungen und Mädchen waren in allen Altersgruppen nahezu gleich häufig betroffen. Die häufigsten Komorbidität bei dieser Kohorte umfasste atopische Erkrankungen (z. B. atopische Dermatitis mit 15,65 %) sowie Pruritus (4,8 %) und Fieber sonstiger und unklarer Genese (10,45 %) [11].

Als leitliniengerechte Therapieempfehlungen sind Antihistaminika der 2. Generation einzusetzen. Erstaunlicherweise wurde bei 25 % der verordneten Antihistaminika Dimetinden rezeptiert, ein Antihistaminikum der 1. Generation. Bereits in der Leitlinie von 2010 wurde die Empfehlung zur Therapie mit Antihistaminika der zweiten Generation ausgesprochen [13]. Dies deckt sich mit aktuellen Daten, die zeigen, dass auch entgegen den aktuellen Leitlinienempfehlungen immer noch vermehrt Antihistaminika der 1. Generation verschrieben werden [8, 14]. Etwa 20 % der Versicherten wurden mit Kortikosteroiden behandelt, mehr als 10 % mit systemischen. Atopische Erkrankungen als Komorbidität traten bei Kindern mit Urtikaria häufiger auf als in der Kontrollgruppe (16,0 % vs. 8,0 %), ebenso Autoimmunerkrankungen, psychologische Probleme und Adipositas [11].

Die hohe Anzahl an verordneten topischen/inhalativen Kortisonpräparaten könnte auch durch die Komorbidität (z. B. Asthma, Neurodermitis) zu erklären sein [11]. Ähnliches gilt auch für die hohe Anzahl verordneter Antibiotika, die sich durch die Komorbidität „Fieber sonstiger und unbekannter Ursache“ erklären ließe [11].

Auch die zunächst zurückhaltende Verordnung systemischer Medikation im Vergleich zur topischen im 1. Quartal nach Erkrankung (Abb. 1) deutet auf einen restriktiven Therapieansatz hin, der sich womöglich aus fehlenden standardisierten Therapieoptionen ergibt.

Das Management der Urtikaria beinhaltet v. a. eine stadiengerechte Therapie. Der Goldstandard sind leitliniengerecht bei allen Altersklassen inklusive Kindern Antihistaminika der 2. Generation. Bei fehlender Symptomkontrolle unter der Einfachdosis von Antihistaminika empfiehlt sich bei Erwachsenen eine Aufdosierung bis auf die 4fache Menge der zugelassenen Tagesdosis, dies gilt gewichtsadaptiert auch für Kinder. Von dieser Aufdosierung profitieren aber nur etwa 50 % der Erwachsenen [1, 6, 7]. Daten zu Kindern fehlen. Seit 2012 ist bei einer ungenügenden Krankheitskontrolle und dem nicht ausreichenden Ansprechen auf hoch dosierte Antihistaminika bei chronisch spontaner Urtikaria (CSU) eine Therapie mit Omalizumab empfohlen. Das Medikament ist seit 2014 ab einem Alter von 12 Jahren für die chronisch spontane Urtikaria zugelassen [14]. Die Tatsache, dass das Medikament seit 2014 für die chronische spontane Urtikaria ab dem 12. Lebensjahr zugelassen ist, sich aber in der Verschreibungsliste von 2015 nicht wiederfindet, deutet auf einen restriktiven Therapieansatz hin.

Eine epidemiologische Studie aus Spanien aus dem Jahr 2004 mit ca. 5000 Befragten, darunter 0,6 % mit chronischer Urtikaria, zeigte, dass etwa 11 % der Patienten mit CSU auch nach 5 Jahren noch an Urtikariasymptomen leiden [5]. Dies bezog sich auf einen Zeitraum, in dem noch keine Biologikatherapie für Urtikaria existierte.

Urtikaria ist eine Krankheit, die eine systemische Therapie erfordert und frühzeitig behandelt werden sollte.

Diese Daten zeigen, dass häufig ein restriktiver Therapieansatz verfolgt wird. Da der Kinderarzt auch heute noch die erste Anlaufstelle ist, sollte ein interdisziplinärer Austausch stattfinden, um ein einheitliches Therapieverfahren zu gewährleisten.

Als nächster Schritt werden nun aktuelle epidemiologische Daten zur Urtikaria bei Kindern analysiert. Hierbei wird es interessant aufzuzeigen, inwieweit sich in Anbetracht der neuen Therapiemöglichkeiten sowohl die Art der verschriebenen Medikamente, in etwa die des Kortisons, aber auch Komorbiditäten, wie z. B. Asthma und Neurodermitis, Krankenhausaufenthalte und Facharztbesuche verändern. Aktuelle Studien sowie die geplante Zulassung von Dupilumab zur Behandlung der Urtikaria bei Kindern ab dem 12. Lebensjahr versprechen neue effektive Therapiemöglichkeiten [12].

Grundlegende Stärke dieser Arbeit ist die im Vergleich zu Primärerhebungen große Stichprobe von mehr als 150.000 Kindern mit und ohne Urtikariadiagnose. Bisher fehlt es an grundlegenden Informationen zur Epidemiologie wie auch Versorgung und zu Inanspruchnahmen von Kindern und Jugendlichen mit Urtikaria. Die Ergebnisse müssen im Kontext einiger Limitationen diskutiert werden.

Bei der Interpretation der Ergebnisse zur Arzneimitteltherapie ist zu berücksichtigen, dass Informationen zu Arzneimitteln fehlen, die ohne Rezept in der Apotheke erworben wurden (Over-the-Counter [OTC]). Eine weitere wichtige Einschränkung der Kassendaten ist, dass keine klinischen Informationen vorliegen und es sich nicht um eine Zufallsstichprobe der deutschen Wohnbevölkerung handelt. Um den Unterschieden zwischen den Krankenkassen Rechnung zu tragen, wurden die administrativen Prävalenzdaten alters- und geschlechtsstandardisiert.

Die Daten liefern erstmals relevante Informationen zu Kennzahlen sowie zur Inanspruchnahme spezifischer Leistungen bei Kindern mit Urtikaria. Diese Erkenntnisse bestätigen sich auch heute noch in unserem klinischen Alltag. Sie bilden somit eine wichtige Grundlage für die Bedarfsplanung im Gesundheitswesen.

## Fazit für die Praxis

- Die vorliegenden Daten unterstreichen die Bedeutung der Urtikaria bei Kindern als typische Hauterkrankung im Kindesalter.
- Die Versorgung ist in Abhängigkeit von der behandelnden Arztgruppe sehr unterschiedlich und bislang noch wenig standardisiert und unzureichend.
- Interdisziplinäre Leitlinien- und Implementierungsprogramme sowie Fortbildungsprogramme auch für Pädiater und Allgemeinmediziner sind hier in Deutschland verstärkt zu fordern.

### Supplementary Information

Tabellen mit den erhobenen Daten
