# Supplementary material for: Care and medical costs of urticaria in children in Germany: Drugs, medical and inpatient services
Source: Dermatologie (Heidelb). 2024 May 22;75(7):554–61. [Article in German] doi: 10.1007/s00105-024-05346-3 (PMC11224104; doi:10.1007/s00105-024-05346-3)
Supplement: Supplementary file 1 — Tabellen mit den erhobenen Daten [file 105_2024_5346_MOESM1_ESM.pdf]

Online-Tabellen 1-5, Versorgung und Krankheitskosten der Urtikaria bei Kindern in Deutschland: Arzneimittel, ärztliche und stationäre Leistungen

Online-Tabelle 1 Berücksichtigte ATC-Kodierungen der relevanten Arzneimittel bei juveniler Urtikaria.

ATC	Medikament	Systemisch	Topisch
J01	Antibiotika zur systemischen Anwendung	X	
R06AA	Antihistaminika zur systemischen Anwendung	X	
D04AA	Antihistaminika zur topischen Anwendung		X
J01C	Betalactam-Antibiotika, Penicilline	X	
A01AC05	Betamethason	X	
R06AX29	Bilastin	X	
R06AE07	Cetirizin	X	
A02BA01	Cimetidin	X	
R06AX27	Desloratadin	X	
A01AC02	Dexamethason		X
D07AB19			X
D07XB05			X
D10AA03			X
H02AB02		X	
R01AD03			X
S01BA01			X
S01CB01			X
S02BA06			X
S03BA01			X
R01AD53			X
D07CB04	Dexamethason und Antibiotika		X
S01CA01	Dexamethason und Antiinfektiva		X
S02CA06			X
S03CA01			X
R06AB03	Dimetinden	X	
R06AX22	Ebastin	X	
R06AE09	Levocetirizin	X	
D07AA01	Methylprednisolon		X
D10AA02			X
H02AB04		X	
H02BX01		X	
D07CA02	Methylprednisolon und Antibiotika		X
S01CA08	Methylprednisolon und Antiinfektiva		X
D07AC14	Methylprednisolonaceponat		X
R03DX05	Omalizumab	X	
A01AC04	Prednisolon		X
A07EA01		X	
D07AA03			X
D07XA02			X
H02AB06		X	
R01AD02			X
S01BA04			X
S01CB02			X
S02BA03			X
S03BA02			X
A02BA02	Ranitidin	X	
R06AX28	Rupatadin	X	

Online-Tabelle 2 Berücksichtigte ATC-Kodierungen der relevanten Arzneimittel bei juveniler Urtikaria in Kombinationstherapie und als Monotherapie für therapieresistente Patienten.

ATC	Arzneimittel	Systemisch	Topisch
**Kombinationstherapie**			
Nicht sedierendes Antihistaminikum			
R06AX27	Desloratadin		
R06AE09	Levocetirizin		
R06AX28	Rupatadin		
R06AX22	Ebastin		
R06AX25	Mizolastin		
R06AX29	Bilastin		
R06AX13	Loratadin		
R06AE07	Cetirizin		
R06AX26	Fexofenadin		
Nicht sedierendes Antihistaminikum in Verbindung mit			
L04AD01	Ciclosporin A		
R03DC03	Montelukast		
A02BA01	Cimetidin		
A14AA02	Stanazolol		
R03DC01	Zafirlukast		
L04AA06	Mycophenolatmofetil		
R06AX17	Ketotifen and Nimesulid		
S01GX08			
M01AX17			
M02AA26			
D07AD01	Clobetasolpropionat		
D07CD01			
D07AB01			
S01BA09			
S01CA11			
**Monotherapie**			
D04AX01	Dopexin		X
N06AA12		X	
R06AX17	Ketotifen	X	
S01GX08			X
P01BA02	Hydroxychloroquin	X	
D10AX05	Dapson		X
J04BA02		X	
A07EC01	Sulfasalazin	X	
L01BA01	Methotrexat	X	
L04AX03		X	
R06AX02	Cyproheptadin	X	
G03XA01	Danazol	X	
R01AA15			X

Online-Tabelle 3 Anteil der Facharztgruppen an der Versorgung für Kinder und Jugendliche mit und ohne eine Urtikariadiagnose im Jahr 2015.

**Facharztgruppe**	**Anzahl Versicherte (n = 1.904)**	**Anzahl Versicherte ohne Urtikaria (n = 149.344)**
	n (%)	n (%)
Pädiatrie	1.349 (70,85)	81.531 (54,59)
Allgemeinmedizin	999 52,47)	67.014 (44,87)
Dermatologie	629 (33,04)	20.469 (13,71)
Hals-Nasen-Ohrenheil-kunde	382 (20,06)	20.839 (13,95)
Innere Medizin	94 (4,94)	3.884 (2,60)

Da die Kinder bei mehreren Ärzten untersucht worden sein können, sind Mehrfachnennungen möglich.

Online-Tabelle 4 Jährliche Kosten je Kind und Jugendlicher mit (n = 1.904) und ohne (n = 149.344) Urtikaria für das Jahr 2015.

		Versicherter mit mind. einer Leistung	Verordnung	Gesamtkosten (€)	Kosten je Versicherten (€)	Kosten je Versicherten und mind. einer Leistung (€)
**Mit Urtikaria**						
**Arzneimittelkosten**		1.387	3.123	48.719,89	25,59	35,13
Arzneimittelgruppe	Systemisch	1.333	2.900	45.703,89	24,00	34,29
	Topisch	199	223	3.015,55	1,58	15,15
Geschlecht	Männlich	625	1.433	22.955,08	12,06	36,73
	Weiblich	762	1.690	25.763,36	13,53	33,81
**Ohne Urtikaria**						
**Arzneimittelkosten**		43.097	146.072	1.618.922,25	10,84	37,56
Arzneimittelgruppe	Systemisch	41.133	89.956	1.542.199,85	10,33	37,49
	Topisch	3.819	3.393	76.772,40	0,51	20,09
Geschlecht	Männlich	20.817	35.082	810.984,49	5,43	38,96
	Weiblich	22.280	38.414	807.937,76	5,41	36,26

Online-Tabelle 5 Arzneimittelverordnungen Antihistaminika in Arzneimittelkombinationen und Monotherapie bei Kindern und Jugendlichen mit (n = 1.387) und ohne (n = 43.097) Urtikaria und einer Arzneimittelverordnung im Jahr 2015.

Arzneimittel	Versicherte n (%)	Verordnungen	DDD	Verordnungen pro Versicherten	DDD pro Versicherten
**Mit Urtikaria**					
***Nichtsedierendes Antihistaminikum in Kombination* gesamt**	**38 (2,74)**	**199**	**8.930**	**5,24**	**235**
Montelukast	31 (2,24)	181	8.524	5,84	275
Ketotifen and Nimesulid	5 (0,36)	13	258	2,60	52
Clobetasolpropionat	2 (0,14)	5	148	2,50	74
Monotherapie - gesamt	**60 (4,33)**	**65**	**2.482**	**1,08**	**41**
Dopexin	51 (3,68)	56	2.280	1,10	45
Ketotifen	8 (0,58)	8	195	1,00	24
Cyproheptadin	1 (0,07)	1	7	1,00	7
**Systemisch**	**4 (7,14)**	**4**	**64**	**1,00**	**16**
**Topisch**	**56 (93,33)**	**61**	**2.418**	**1,09**	**43**
**Ohne Urtikaria**					
***Nichtsedierendes Antihistaminikum in Kombination* gesamt**	**361 (0,84)**	**1.402**	**55.971**	**3,88**	**155**
Montelukast	250 (0,58)	1.068	46.241	4,27	185
Ketotifen and Nimesulid	98 (0,23)	274	7.439	2,80	76
Clobetasolpropionat	19 (0,04)	60	2.291	3,16	121
Monotherapie - gesamt	**1.509 (3,50)**	**1.743**	**45.678**	**1,16**	**30**
Dopexin	1.169 (2,71)	1.336	36.068	1,14	31
Ketotifen	340 (0,79)	405	9.598	1,19	28
Cyproheptadin	1 (0,00)	1	33	1,00	33
**Systemisch**	**70 (4,64)**	**103**	**1.500**	**1,47**	**21**
**Topisch**	**1.441 (95,49)**	**1.640**	**44.178**	**1,14**	**31**

DDD, defined daily dose (definierte Tagesdosis).
